# Auricular Acupuncture May Suppress Epileptic Seizures via Activating the Parasympathetic Nervous System: A Hypothesis Based on Innovative Methods

**DOI:** 10.1155/2012/615476

**Published:** 2012-02-01

**Authors:** Wei He, Pei-Jing Rong, Liang Li, Hui Ben, Bing Zhu, Gerhard Litscher

**Affiliations:** ^1^Institute of Acupuncture and Moxibustion, China Academy of Chinese Medical Sciences, Beijing 100700, China; ^2^Stronach Research Unit for Complementary and Integrative Laser Medicine, TCM Research Center Graz, and Research Unit of Biomedical Engineering in Anesthesia and Intensive Care Medicine, Medical University of Graz, Auenbruggerplatz 29, 8036 Graz, Austria

## Abstract

Auricular acupuncture is a diagnostic and treatment system based on normalizing the body's dysfunction. An increasing number of studies have demonstrated that auricular acupuncture has a significant effect on inducing parasympathetic tone. Epilepsy is a neurological disorder consisting of recurrent seizures resulting from excessive, uncontrolled electrical activity in the brain. Autonomic imbalance demonstrating an increased sympathetic activity and a reduced parasympathetic activation is involved in the development and progress of epileptic seizures. Activation of the parasympathetic nervous system such as vagus nerve stimulation has been used for the treatment of intractable epilepsy. Here, we propose that auricular acupuncture may suppress epileptic seizures via activating the parasympathetic nervous system.

## 1. Introduction

Epilepsy is a neurological disorder consisting of recurrent seizures resulting from excessive, uncontrolled electrical activity in the brain. Despite active pharmacological and neurosurgical treatments used for the treatment of epileptic disorders, the management of medically intractable epilepsy remains a difficult problem.

Over the past two decades, concerns regarding the side effects of pharmacological and neurosurgical approaches have increased interest in the use of complementary and alternative medicine (CAM) [1–3].

Autonomic imbalance is involved in the development and progress of epileptic seizures. Auricular acupuncture can treat diseases by increasing parasympathetic tone. Here, we propose that auricular acupuncture may suppress epileptic seizures via activating the parasympathetic nervous system.

## 2. Auricular Acupuncture Can Increase Parasympathetic Tone

Auricular acupuncture is a diagnostic and treatment system based on normalizing the body's dysfunction which is suggested to stimulate the peripheral reflexes, then activate these central brain pathways, and thus inhibit the maladaptive reflexes that contribute to neuropsychic disorders [4]. Auricular acupuncture was utilized to treat postoperative pain [5], improve neurorehabilitation [6], insomnia [7], and obesity [8] via modifying endorphinergic systems and the autonomic nervous system (ANS). 

An increasing number of studies have demonstrated that auricular acupuncture has a significant effect on inducing parasympathetic tone. Manual ear acupressure at “heart” auricular acupoint induced a significant decrease in heart rate and a significant increase in heart rate variability total [9, 10]. Acupuncture on auricular acupoint “Shenmen” might calm the mind, slow down the heart rate, activate the parasympathetic nerves, and inhibit the sympathetic nerves [11]. Acupuncture conducted on the concha of the ear induces an increase in vagal activity [12]. During needling vision-related acupoints of ear acupuncture, mean blood flow velocity of the ophthalmic artery was significantly increased which may be induced by parasympathetic tone [13]. Another clinical study showed that stimulation of the ear induced a significant increase in the parasympathetic activity during the stimulation period of 25 min and during the poststimulation period of 60 min [14]. The external ear is innervated by several nerves, including vagus nerve, glossopharyngeal nerve, trigeminal nerve, facial nerve, and branches (the second and third) of the cervical spinal nerves [15]. The auricular branch of vagus nerve (ABVN) innervates the auricular concha and the external auditory meatus. Parasympathetic tone such as Arnold's reflexes has been clinically observed after stimulating innervation regions of the ABVN [16, 17], which is considered as a bridge between the external ear and the internal organs [18]. In Traditional Chinese Medicine, auricular acupoints related to internal organs are located at the auricular concha [4]. Except for the ABVN, the glossopharyngeal nerve, the trigeminal nerve, and the facial nerve, all carry parasympathetic nerve fibers. Most nerves innervating the external ear carry parasympathetic components.

## 3. Epilepsy Is Associated with Decreased Parasympathetic Tone

Autonomic symptoms accompany all generalized tonic-clonic seizures (GTC) and one-third of simple partial seizures. The ANS centers can be involved in complex partial, absence, and generalized tonic seizures. Measurements of ANS functions may be helpful in differentiating between epileptic seizures and nonepileptic psychogenic seizures [19]. The autonomic imbalance of epileptic seizures probably results from the hypersynchronized electrical impulse from the temporal and frontal areas to the limbic system, then to autonomic central nuclei in medulla including the nucleus tractus solitarius (NTS) and ambiguus nuclei. Both sympathetic and parasympathetic efferent discharges are then generated.

There is ample experimental and clinical proof that epilepsy goes along with autonomic imbalance demonstrating an increased sympathetic activity and a reduced parasympathetic activation. Novak et al. documented rapid parasympathetic withdrawal approximately 30 seconds before seizure onset and a sympathetic activation peak at seizure onset [20]. Temporal lobe epilepsy is known to be associated with ictal and interictal autonomic dysregulation, predominantly with sympathetic overactivity [21]. Higher sympathetic function and lower parasympathetic function have been demonstrated to be significant risk factors for sudden unexplained death in epilepsy subjects [22, 23].

Activation of the parasympathetic nervous system (PNS) has shown therapeutic benefits in brain diseases. Examples include vagus nerve stimulation (VNS) for epilepsy. VNS has been successfully applied for more than 20 years to treat drug-resistant epilepsy [24]. The antiseizure effect of VNS is considered to be mediated via vagal afferent projections to the NTS, then from the NTS to different brain regions which correlate with the pathogenesis of epilepsy [25]. Recently, VNS has also been applied for treatment of drug-resistant depression [26] and was suggested as a new approach for the treatment of heart failure [27] and stroke [28] by increasing the parasympathetic tone.

## 4. Hypothesis

Auricular acupuncture appears to modify the autonomic dysfunction by increasing parasympathetic activity. Thus, we hypothesize that auricular acupuncture may suppress epilepsy by increasing parasympathetic tone. We have done clinical trials and animal experiments on the effect and mechanism of auricular electroacupuncture for the treatment of epilepsy. In clinical trials, auricular electroacupuncture reduced seizure frequency and attenuated seizure severity. Animal results showed that auricular electroacupuncture suppressed epileptic discharges in electroencephalogram traces. All the results support our hypothesis.

## 5. The Mechanism of Auricular Acupuncture for Epilepsy

**Figure 1:**
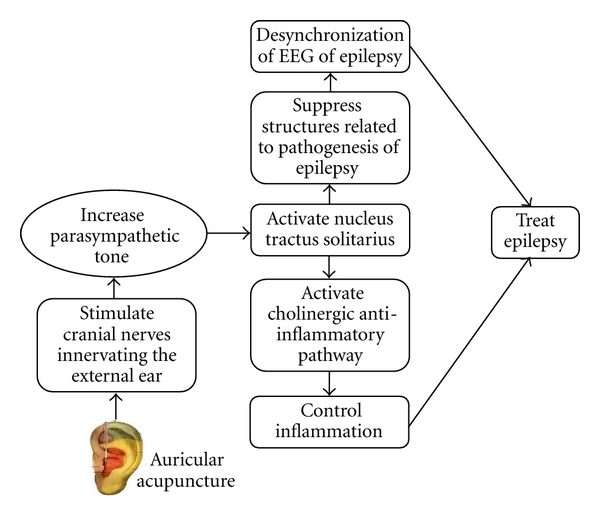
Possible mechanism of auricular acupuncture for the treatment of epilepsy.

Acupuncture has been used to treat epilepsy. Acupoints selected to treat epilepsy included “GV 14” [29, 30], “ST 36” [31], and auricular acupoints such as “Pizhixia, Nao, and Shenmen” [32, 33]. Most nerves innervating the external ear carry parasympathetic nerve fibers. Moreover, the ABVN is the only peripheral branch of the vagus nerve. Acupuncture at auricular acupoints especially in the area of auricular concha may induce vagal tone to suppress epileptic seizures. As the main vagal afferent, the NTS is considered as a neuroanatomical center for pathways of the antiseizure effect of auricular acupuncture [34]. Amelioration of illness by auricular acupuncture is believed to be through the reticular formation which is found to be histopathologically connected with focal-cortical seizure-induced generalized convulsive status epilepticus [35]. Recent findings highlight the possibility of inflammation in seizures and epileptogenesis [36]. Prototypical inflammatory cytokines such as IL-1*β*, TNF-*α*, and IL-6 have been shown to be overexpressed prominently by glia. Cytokines receptors are also upregulated, and the related intracellular signalling is activated in brain areas of seizure generation and propagation in experimental models of seizures [37]. The anti-inflammation effect perhaps is the mechanism of auricular acupuncture for epilepsy [38]. Possible mechanism of auricular acupuncture for the treatment of epilepsy is shown in Figure 1.

## 6. Potential Application of Auricular Acupuncture for Other Diseases

The ANS is the primary neural mediator of physiological responses to internal and external stimuli [39]. Functions of many or perhaps all visceral organs can be modulated by somatosympathetic or somatoparasympathetic reflex activity induced by an appropriate somatic afferent stimulation [40]. According to the theory of Traditional Chinese Medicine, acupuncture has the function of bidirection-regulative effect. In addition to the dysfunction of parasympathetic system, auricular acupuncture can also modulate the dysfunction of sympathetic system [41]. Therefore, auricular acupuncture provides a somatic stimulation to treat diseases being induced or accompanied by an imbalance of the autonomic system.

VNS has been proposed to have the potential for the treatment of neuropsychiatric illnesses [42]. Yet it is an invasive procedure that may have potential side effects and complications. Its application in developing countries is limited for high costs. We can use auricular electroacupuncture, which is less invasive, of less cost, and convenient to treat diseases by setting up suitable parameters. Recently, kinds of acupuncture treatment instruments such as radio electric stimulator device [43] and P-Stim auricular electroacupuncture stimulation device [44] have been developed for the treatment of stress-related disorders and pain relief. Auricular vagal nerve stimulator is expected to be explored.

## Abbreviations

CAM: Complementary and alternative medicine
GTC: Generalized tonic-clonic seizures
ANS: Autonomic nervous system
NTS: Nucleus tractus solitarius
VNS: Vagus nerve stimulation
PNS: Parasympathetic nervous system
ABVN: Auricular branch of vagus nerve.
